# Speed of Sound Measurements of R-1130(E) and an Azeotropic Blend of R-1336mzz(Z)/1130(E)

**DOI:** 10.1007/s10765-024-03416-1

**Published:** 2024

**Authors:** Aaron J. Rowane

**Affiliations:** 1 Applied Chemicals and Materials Division, National Institute of Standards and Technology, Boulder, CO 80305, USA

**Keywords:** R-1130(E), R-1336mzz(Z), R-514A, REFPROP, Refrigerant mixtures, Speed of sound

## Abstract

Sound speed data measured using a dual-path pulse-echo instrument are reported for pure *trans*-1,2-dichloroethene (R-1130(E)) and an azeotropic blend of *cis*-1,1,1,4,4,4-hexafluorobutene (R-1336mzz(Z)) and R-1130(E) with a composition of 74.8 mass % R-1336mzz(Z) with the balance being R-1130(E). The azeotropic blend of R-1336mzz(Z)/1130(E) is classified as R-514A in ANSI/ASHRAE standard 34. Liquid phase speed of sound data are reported from just above the saturation pressure of pure R-1130(E) or the bubble point pressure of R-514A to a maximum pressure of 26.7 MPa. The relative combined expanded uncertainty in the speed of sound varies from 0.032 % to 0.148 % with the greatest deviations occurring at the lowest sound speeds. At present, no reference Helmholtz-energy-explicit equation of state (EOS) is available for R-1130(E). Therefore, the reported data for pure R-1130(E) are compared to an extended corresponding states (ECS) model. Deviations between the pure R-1130(E) sound speed data and the ECS model were found to be consistently negative ranging between − 4.1 % and − 3.5 %. The R-514A data are compared to a multifluid model inclusive of the established reference Helmholtz-energy-explicit EOS for R-1336mzz(Z) and ECS model for R-1130(E) with estimated binary interaction parameters. Deviations between the experimental speed of sound data and the multifluid model were also found to be consistently negative. However, deviations from the multifluid model were found to be as great as − 17.1 %. The large deviations from the ECS model and multifluid model underscore the need for a robust Helmholtz-energy-explicit EOS for R-1130(E).

## 1 Introduction

The azeotropic blend of *cis*-1,1,1,4,4,4-hexafluorobutene (R-1336mzz(Z)) and *trans*-1,2-dichloroethene (R-1130(E)) is classified as R-514A in ANSI/ASHRAE standard 34 [1] with a composition of 74.7 mass % R-1336mzz(Z) with the balance being R-1130(E). R-514A was found to be a suitable replacement for the high global warming potential refrigerant 2,2,-dichloro-1,1,1-trifluoroethane (R-123) used in low-pressure centrifugal chillers. R-514A relative to R-123 offers a 97 % reduction in global warming potential. Both R-514A and R-123 offer zero temperature glide and comparable performance. While an abundance of R-1336mzz(Z) thermophysical property data are available in the literature, only a handful of studies report thermophysical properties for R-1130(E). Further, extremely limited thermophysical property data are available for mixtures of R-1336mzz(Z) and R-1130(E). The efficient optimization of refrigeration equipment requires the availability of thermophysical properties of working fluids.

In previous studies by our group we report vapor–liquid equilibria (VLE) data for R-1336mzz(Z) + R-1130(E) blends [2] and the thermal conductivity of R-1130(E) [3]. In this study, this effort is expanded reporting sound speed data measured using a dual-path pulse-echo instrument for pure R-1130(E) and a blend very close to R-514A with a composition of 74.8 mass % R-1336mzz(Z) with the balance being R-1130(E). Further, density measurements are planned for R-1130(E). These data will support the development of a reference equation of state (EOS) for R-1130(E) and a multifluid model for the R-1336mzz(Z) + R-1130(E) mixture. Accomplishing this goal is contingent on the availability of robust Helmholtz-energy-explicit EOS for each pure component and binary interaction parameters fit to reference quality mixture data. Ideally, the reference data should be for several thermodynamic properties including VLE, density, and speed of sound. McLinden and Akasaka [4] summarize the literature data for R-1336mzz(Z) and report a reference EOS for the fluid which was developed using reference quality vapor pressure, density, and speed of sound data. Our previous work summarizes the available vapor pressure data for R-1130(E) [2], which were found to be abundant although significant scatter in the data of various literature sources was observed. Fewer density data sets are available for R-1130(E) [5–7]. Presently, no study in the literature reports speed of sound data for R-1130(E) over a wide range of state points. The pure R-1130(E) data reported in this study is compared to the extended corresponding states (ECS) model applied by Tanaka et al. [7], which is a general ECS model for refrigerants developed by Teraishi et al. [8]

Mixture data for R-1336mzz(Z) + R-1130(E) are limited to VLE data reported by our group [2] and a single isothermal data set reported in the application to add R-514A to ANSI/ASHRAE standard 34 [1]. The mixture data are compared to a multifluid model incorporating the reference EOS of McLinden and Akasaka [4] for R-1336mzz(Z) and the ECS model for R-1130(E) with binary interaction parameters estimated by REFPROP version 10.0 [9].

## 2 Materials and Methods

The samples used in this study are identical to those used in our previous study [2] and therefore only limited information is restated here. The dual-path pulse-echo instrument and isochoric measurement procedure used in this study are identical to those reported in previous studies [10–13]. Therefore, only brief descriptions of the instrument and measurement procedure are provided here, and the reader is referred to our previous work for more detail.

### 2.1 Sample Preparation

Table 1 lists the refrigerants used in this study along with their short names, CAS number, source, and purity. Prior to preparing mixtures each refrigerant listed in Table 1 was degassed using a freeze/pump/thaw technique to remove any volatile impurities. Further information on the analysis techniques to quantify their sample purities can be found in our groups previous publications [2, 4]. In this work, the vapor pressure of the pure refrigerants was significantly lower than those from previous studies. Therefore, in contrast to previous studies with refrigerants [12, 13] a liquid-phase mixture rather than a vapor phase mixture was prepared to load our instrument as significant fractionation of the sample during storage was not a concern. The R-514 blend had a composition of 74.8 mass % R-1336mzz(Z) with the balance being R-1130(E). The uncertainty in the composition of the R-514A blend was 0.002 mass fraction. The R-1336mzz(Z)/1130(E) mixture used in this study is identical to that from our previous study [2] and the interested reader is directed there for more information on the mixture preparation procedure.

### 2.2 Dual-Path Pulse-Echo Instrument

The dual-path pulse-echo instrument used in this study could operate at temperatures ranging from 230 K to 423 K and was capable of measurements to pressures of 93 MPa. The temperature and pressure measurements had standard uncertainties of 0.004 K and 0.014 MPa, respectively. Relative to refrigerants measured in previous studies the refrigerants studied here have significantly higher boiling points and critical points and therefore the relative combined expanded uncertainty of the speed of sound for the R-1130(E) measurements ranged from 0.032 % to 0.053 % with an average uncertainty of 0.038 %. For the R-514A blend the uncertainties were slightly higher ranging from 0.035 % to 0.148 % with an average uncertainty of 0.053 %. The relative combined expanded speed of sound uncertainties, *U*_r_(*w*), were determined with a coverage factor, *k* = 2. The uncertainty analysis considered contributions from the sample composition, temperature, pressure, time delay between echo arrivals, and path length calibration. Uncertainties were calculated using the analysis presented in our groups previous publications[10, 12]. Unlike previous studies the speed of sound was measured at nearly the full range of temperatures that the instrument is capable of (230 K to 420 K) as the echo signal strength remained strong enough for sensitive speed of sound measurements up to 420 K.

### 2.3 Loading and Isochoric Measurement Procedure

The measuring cell, manifold, and filling line were evacuated for 12 h at a vacuum pressure of 8·10^−4^ Pa before loading any sample. The liquid samples for both pure R-1130(E) and the R-514A blend were then loaded into the measuring cell. To ensure that the measuring cell was filled with sufficient sample to perform measurements over a wide range of state points the system was first cooled to 228 K. The sample was heated using a heat lamp to increase the sample temperature and its vapor pressure so that the sample temperature exceeded the temperature of the manifold (296 K). This ensured that both the measuring cell and manifold were filled with liquid. Once the system was filled with liquid the measurements commenced starting from a temperature of 230 K for R-1130(E) and 240 K for the R-514A blend. For measurements along isochores the temperature was increased in 5 K increments until the pressure was about 25 MPa. For pure R-1130(E) isochores were typically chosen to start at a temperature 10 K higher than that of the previous isochore. For the R-514A blend, isochores were typically chosen to start at a temperature 20 K higher than that of the previous isochore. Measurements were performed on both samples to a maximum temperature of 420 K. A more detailed description of the isochoric measurement procedure can be found in our previous study [12].

## 3 Results and Discussion

The following sections present the measured speed of sound data for both pure R-1130(E) and the R-514A blend. The data for pure R-1130(E) is compared to the ECS model of Teraishi et al. [8], which is a generalized model for refrigerants that was recently applied by Tanaka et al. for R-1130(E) [7]. The data for the R-514A blend is compared to a multifluid model incorporating the ECS model for R-1130(E) and reference equation of McLinden and Akasaka [4] for R-1336mzz(Z) with estimated binary interaction parameters [9].

### 3.1 Experimental Speed of Sound Data

**Fig. 1:**
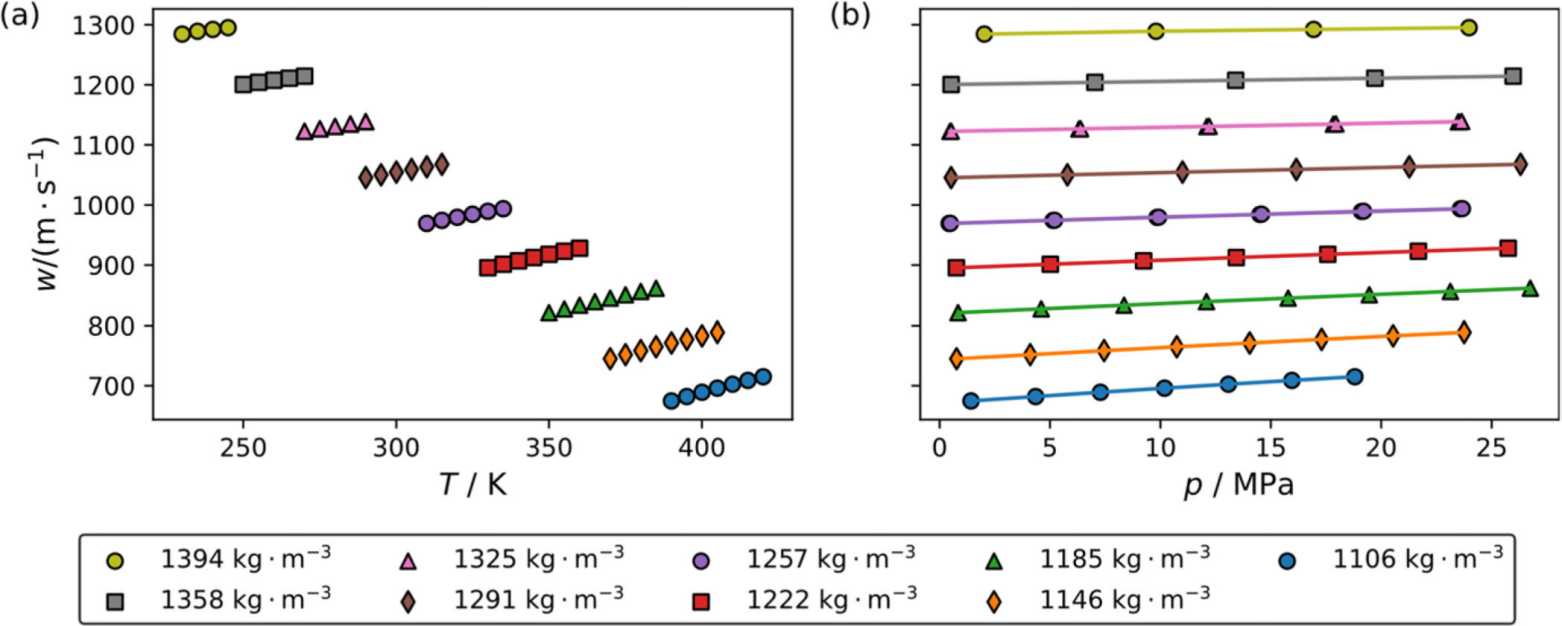
Impact of temperature and pressure on the speed of sound along pseudo-isochores for pure R-1130(E). Symbols represent experimental data points while lines are drawn to guide the eye. To avoid clutter on the graph only every other pseudo-isochore is plotted

**Fig. 2:**
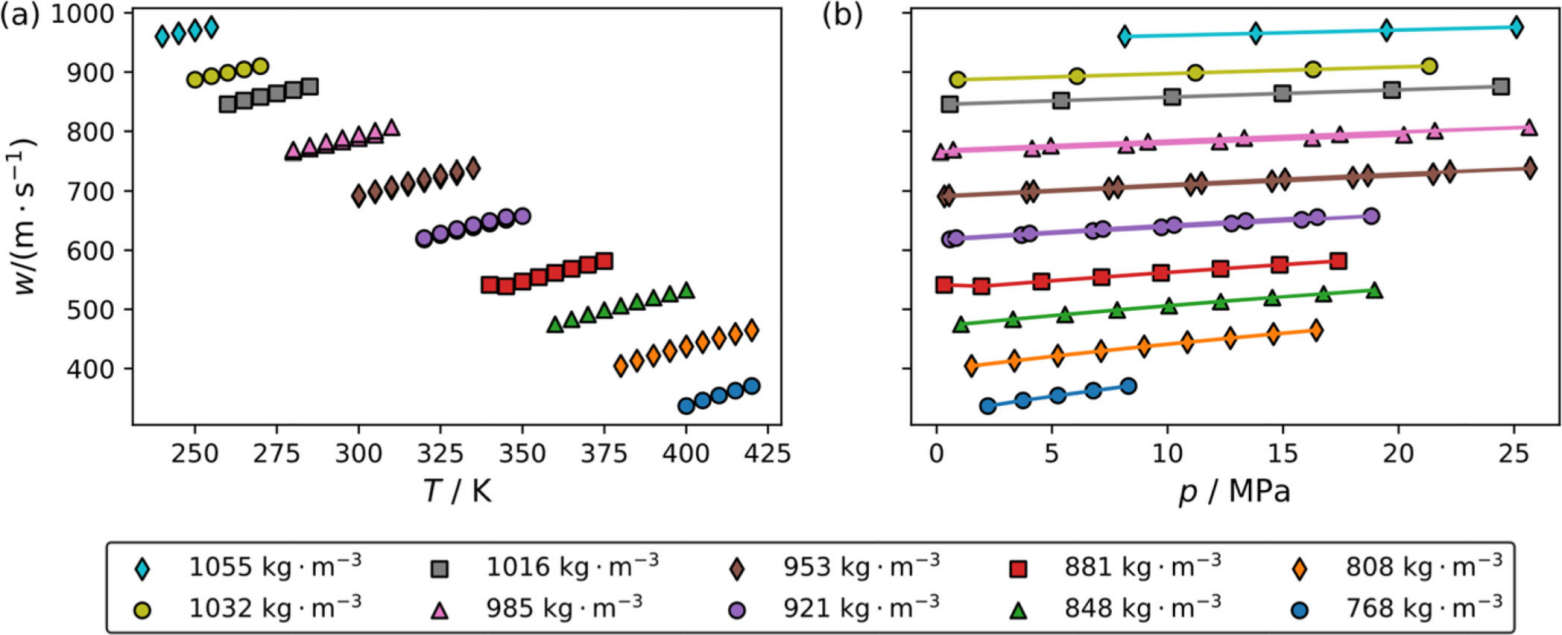
Impact of temperature and pressure on the speed of sound along pseudo-isochores for the R-514A blend, which consists of 74.8 mass % of R-1336mzz(Z) and the balance being R-1130(E). Symbols represent experimental data points while lines are drawn to guide the eye

Figures 1(a) and 1(b) show the impact of temperature and pressure on the speed of sound of pure R-1130(E), respectively. Different colored symbols represent measurements along different pseudo-isochores. The average densities calculated using the ECS model for R-1130(E) are listed next to each symbol in the legend. The plot shows that along each pseudo-isochore the speed of sound is most sensitive to a change in density and less sensitive to changes in temperature and pressure. Every other isochore is plotted in Fig. 1 to avoid clutter on the plot. Figures 2(a) and 2(b) show the impact of temperature and pressure on the speed of sound of the R-514A blend along pseudo-isochores. Densities for each pseudo-isochore were calculated using a multifluid model with the reference equation of McLinden and Akasaka for R-1336mzz(Z) and the ECS model of Teraishi et al. [8] for R-1130(E) with binary interaction parameters estimated by REFPROP version 10.0 [9]. In comparison to pure R-1130(E), the R-514A blend has significantly lower densities and speed of sound values. The changes with temperature and pressure are also slightly more pronounced than those for R-1130(E). Tables 2 and 3 list experimental speed of sound data averaged from up to 12 replicate measurements along with the corresponding temperature, pressure, and measurement uncertainty. To test for sample decomposition, replicate isochore measurements were performed after taking the sample to a temperature of 420 K. In all cases the replicate isochore measurements were well within the stated experimental uncertainty. It is important to note that in Table 2 for pure R-1130(E) the repeat measurements were at virtually the same pressure and temperature and were grouped together. However, for the R-514A data in Table 3 there were slight difference in the temperatures and pressures and the repeat isochores are listed separately. Data listing all the unaveraged speed of sound measurements and their associated uncertaities can be found in the supplementary information, and these data are also deposited at www.nist.data.gov (DOI: https://doi.org/10.18434/mds2-3400).

### 3.2 Comparison to ECS Model for R-1130(E)

**Fig. 3:**
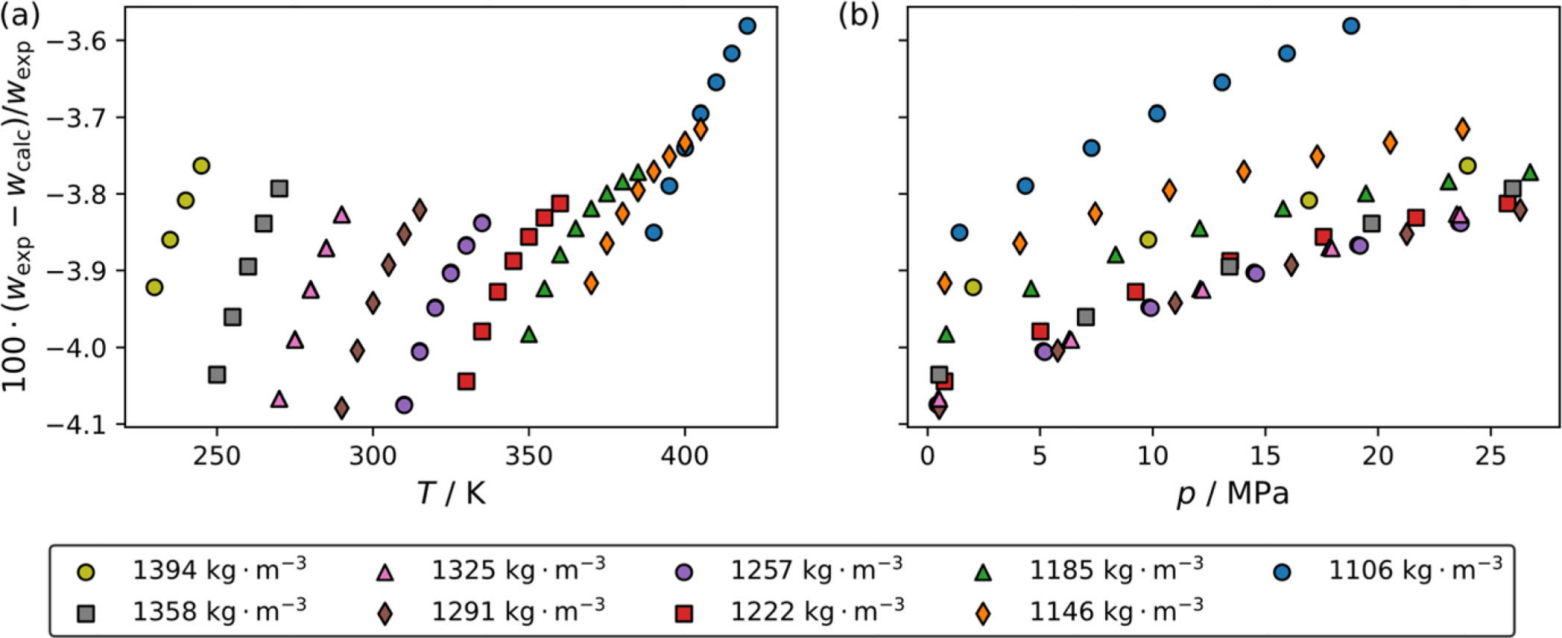
Comparisons of the R-1130(E) speed of sound measurements to the extended corresponding states model of Teraishi et al. [8], as a function of (a) temperature and (b) pressure. Different symbols in the legend represent the different pseudo-isochores

Figures 3(a) and 3(b) present comparisons of the experimental speed of sound data from this study to those calculated using the ECS model for R-1130(E) as a function of temperature and pressure, respectively. Different symbols in the plot represent the different pseudo-isochores. The ECS model is a generalized model for refrigerants developed by Teraishi et al. [8], which was recently applied to R-1130(E) by Tanaka et al. [7] As shown in Fig. 3 the model deviations from experimental data are consistently negative ranging between −4.1 % and −3.5 %. The deviations are seen to decrease with an increase in temperature and pressure. Despite this model not using any speed of sound data for R-1130(E) in its development, it provides a reasonable estimate of the speed of sound for R-1130(E). However, to represent the speed of sound of R-1130(E) more faithfully, a Helmholtz-energy-explicit reference EOS should be developed.

### 3.3 Comparison of the R-514A Blend to the Multifluid Model

**Fig. 4:**
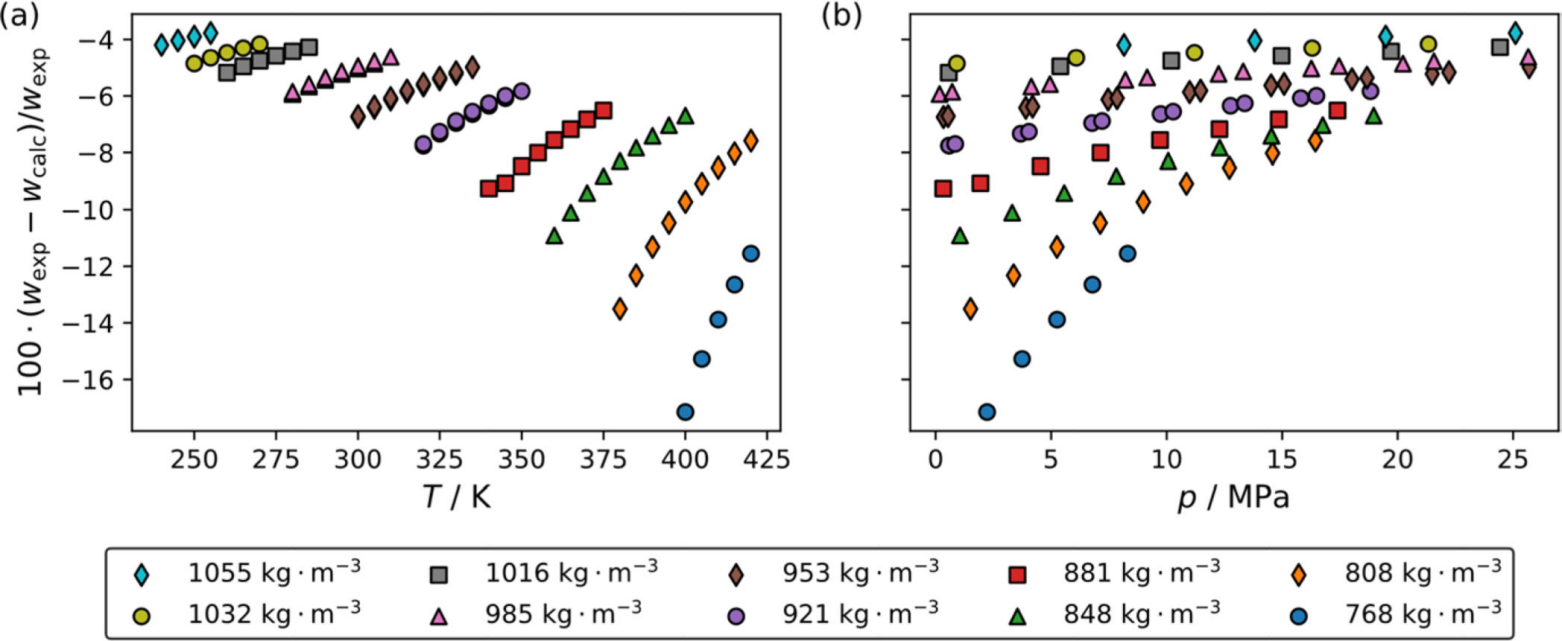
Comparisons of the R-514A blend (74.8 mass % R-1336mzz(Z) and 25.2 mass % R-1130(E)) speed of sound measurements to a multifluid model incorporating the Helmholtz-energy-explicit EOS of McLinden and Akasaka [4] for R-1336mzz(Z) and the extended corresponding states model of Teraishi et al. [8] for R-1130(E) with binary interaction parameters estimated by REFPROP version 10.0 [9], as a function of (a) temperature and (b) pressure. Different symbols in the legend represent the different pseudo-isochores

Figure 4(a) and 4(b) present comparisons with temperature and pressure, respectively, of the experimental data reported in this study to a multifluid model incorporating the Helmholtz-energy-explicit reference EOS of McLinden and Akasaka [4] and the ECS model for R-1130(E) [7, 8]. The binary interaction parameters used to evaluate the multifluid model were estimated by REFPROP version 10.0 [9]. The estimated binary interaction parameters are listed in Table 4. The deviations range from − 3.8 % to − 17.1 %, which are considerably higher than that for pure R-1130(E) in comparison to the ECS model alone. In previous work, calculations with well-established pure fluid equations of state and estimated binary interaction parameters performed within 4 % [11–13]. This underscores the need for a more robust reference EOS for R-1130(E) to provide more accurate predictions for mixtures.

## 4 Conclusions

Liquid phase speed of sound data for R-1130(E) and a blend approximating R-514A are reported at temperatures ranging from 230 K to 420 K and pressures up to 26.7 MPa. These data compliment bubble point and thermal conductivity data reported in our previous studies, which are part of a greater effort to develop a robust Helmholtz-energy-explicit EOS for R-1130(E) and a multifluid model for the R-1130(E)/1336mzz(Z) mixture. The pure R-1130(E) data were compared to the ECS model of Teraishi et al. [8], which was recently applied by Tanaka et al. [7] for R-1130(E). Deviations from the ECS model were found to be consistently negative ranging from −3.6 % to −4.1 %. The speed of sound data for the R-514A blend was compared to a multifluid model that incorporated the reference EOS of McLinden and Akasaka [4] for R-1336mzz(Z) and the ECS model for R-1130(E) using binary interaction parameters estimated by REFPROP version 10.0 [9]. Deviations from the experimental data were found to be significantly greater, ranging from − 3.8 % to − 17.1 %. Further work is planned to measure the density of R-1130(E) with the goal of developing a robust Helmholtz-energy-explicit EOS for the fluid.

## Supplementary Material

UnaveragedSOSData

## Figures and Tables

**Table 1:** Suppliers of pure fluids used in the preparation of mixtures for this work

Chemical	CAS number	Manufacturer	Analysis method	Purity [mole frac]
*cis*−1,1,1,4,4,4-hexafluorobut-2-ene (R-1336mzz(Z))	692-49-9	Chemours	GC/QToF-MS^a^	0.9999[4]
*trans*−1,2-dichloroethane (R-1130(E))	156-60-5	Chemours	NMR^b^	> 0.9910[2]

a Gas-chromatography/quadrupole time-of-flight mass spectroscopy

b Nuclear magnetic resonance

**Table 2:** Experimental speed of sound data for R-1130(E). Speed of sound values listed are averaged from up to twelve measurements at each state point. Isochores are separated by breaks in order of decreasing density. Duplicate points are experimental repeat data points used to confirm sample degradation did not occur. The standard uncertainties for temperature and pressure are *u*_c_(*T*) = 0.004 K and *u*_c_(*p*) = 0.014 MPa, respectively. Relative combined expanded speed of sound uncertainties, *U*_r_(*w*), determined with a coverage factor of *k* = 2 are listed at each state point

*T*/K	*p*/MPa	*w*/m·s^−1^	100·*U*_r_ (*w*)
230.004	2.025	1284.55	0.032
235.016	9.803	1289.25	0.032
240.007	16.936	1292.29	0.032
245.012	23.975	1295.26	0.032
245.008	6.378	1240.16	0.032
250.006	13.306	1244.02	0.032
255.008	20.058	1247.55	0.032
250.019	0.520	1200.48	0.033
255.013	7.029	1204.11	0.033
260.009	13.406	1207.53	0.033
265.014	19.714	1210.90	0.033
270.008	25.971	1214.32	0.033
259.981	0.434	1161.25	0.033
260.000	0.547	1161.52	0.033
264.991	6.630	1165.14	0.033
265.009	6.664	1165.14	0.033
269.987	12.750	1168.98	0.033
270.008	12.721	1168.78	0.033
274.991	18.827	1172.84	0.033
275.013	18.654	1172.18	0.033
279.996	24.814	1176.56	0.033
280.018	24.619	1175.84	0.033
269.987	0.521	1122.66	0.033
270.009	0.467	1122.38	0.033
274.991	6.378	1126.73	0.033
275.013	6.307	1126.40	0.033
279.995	12.200	1130.85	0.033
280.018	12.100	1130.41	0.033
284.999	17.949	1134.85	0.033
285.019	17.833	1134.38	0.033
290.002	23.642	1138.82	0.033
290.019	23.498	1138.26	0.033
279.994	0.461	1083.77	0.034
280.015	0.435	1083.60	0.034
284.998	6.023	1088.14	0.034
285.018	5.985	1087.93	0.034
290.002	11.537	1092.46	0.034
290.020	11.502	1092.28	0.034
295.001	16.994	1096.73	0.033
295.012	16.927	1096.45	0.033
300.004	22.419	1100.99	0.033
300.009	22.309	1100.57	0.033
290.016	0.519	1045.59	0.034
295.012	5.788	1050.23	0.034
300.010	10.998	1054.75	0.034
305.005	16.152	1059.16	0.034
310.005	21.272	1063.54	0.034
314.998	26.303	1067.70	0.034
300.001	0.407	1006.97	0.035
300.007	0.410	1006.97	0.035
305.004	5.395	1011.87	0.035
305.004	5.403	1011.88	0.035
310.003	10.346	1016.68	0.034
310.007	10.360	1016.72	0.034
314.997	15.259	1021.46	0.034
315.000	15.267	1021.47	0.034
320.005	20.140	1026.13	0.034
320.006	20.135	1026.13	0.034
325.008	24.966	1030.70	0.034
325.009	24.966	1030.71	0.034
310.002	0.431	969.14	0.036
310.004	0.480	969.36	0.036
314.995	5.145	974.27	0.035
314.999	5.207	974.54	0.035
320.004	9.845	979.36	0.035
320.005	9.917	979.68	0.035
325.007	14.584	984.71	0.035
325.009	14.503	984.35	0.035
329.998	19.199	989.61	0.035
329.999	19.113	989.23	0.035
335.000	23.670	993.92	0.035
335.003	23.587	993.56	0.035
320.001	0.529	931.85	0.037
320.005	0.431	931.29	0.037
325.006	5.029	937.41	0.036
325.006	4.916	936.80	0.036
329.998	9.475	942.79	0.036
329.998	9.366	942.21	0.036
335.001	13.682	946.99	0.036
335.003	13.810	947.63	0.036
340.005	18.030	951.97	0.035
340.007	18.169	952.64	0.035
345.010	22.356	956.91	0.035
345.012	22.494	957.57	0.035
350.007	26.645	961.77	0.035
330.001	0.756	895.47	0.038
335.003	5.018	901.33	0.037
340.007	9.240	906.98	0.037
345.012	13.432	912.49	0.037
350.009	17.581	917.84	0.036
355.013	21.681	922.96	0.036
359.998	25.744	927.99	0.036
340.002	0.180	854.36	0.039
345.008	2.436	849.77	0.039
350.007	6.364	855.82	0.039
355.012	10.277	861.74	0.038
359.998	14.143	867.46	0.038
365.000	17.989	873.02	0.037
369.992	21.794	878.40	0.037
374.995	25.580	883.66	0.037
350.003	0.831	820.91	0.041
355.008	4.603	827.16	0.040
359.995	8.349	833.28	0.040
364.997	12.081	839.24	0.039
369.992	15.782	845.02	0.039
374.995	19.463	850.66	0.038
380.022	23.134	856.17	0.038
385.013	26.748	861.48	0.037
359.997	1.223	786.14	0.043
364.999	4.782	792.64	0.042
369.992	8.314	798.94	0.041
374.994	11.833	805.06	0.041
380.021	15.344	811.03	0.040
385.012	18.808	816.79	0.039
390.041	22.275	822.45	0.039
395.038	25.695	827.91	0.038
369.986	0.767	745.12	0.046
374.990	4.106	752.01	0.045
380.018	7.445	758.71	0.044
385.010	10.739	765.13	0.043
390.047	14.043	771.41	0.042
395.038	17.297	777.44	0.041
400.046	20.539	783.30	0.041
405.055	23.755	788.96	0.040
380.015	0.960	708.60	0.049
385.008	4.081	715.73	0.048
390.037	7.213	722.66	0.047
395.036	10.305	729.28	0.046
400.046	13.386	735.69	0.045
405.055	16.444	741.87	0.044
410.054	19.477	747.85	0.043
415.071	22.502	753.69	0.042
420.074	25.497	759.34	0.041
390.034	1.420	674.69	0.053
395.033	4.352	682.04	0.051
400.042	7.279	689.14	0.050
405.051	10.188	695.95	0.048
410.051	13.075	702.51	0.047
415.069	15.954	708.84	0.046
420.073	18.808	714.98	0.045
400.041	1.352	635.81	0.059
405.051	4.084	643.46	0.057
410.050	6.800	650.80	0.055
415.068	9.512	657.86	0.053
420.073	12.194	664.58	0.051

**Table 3:** Experimental speed of sound data for the R-514A blend with a composition of 74.8 mass % of R-1336mzz(Z) with the balance being R-1130(E). Speed of sound values listed are averaged from up to twelve measurements at each state point. Isochores are separated by breaks in order of decreasing density. Several isochoric data sets at similar temperatures and pressures are listed and were performed to confirm that the sample did not degrade. The standard uncertainties in temperature, pressure, and composition are *u*_c_(*T*) = 0.004 K, *u*_c_(*p*) = 0.014 MPa, and 0.002 mass fraction, respectively. Relative combined expanded speed of sound uncertainties, *U*_r_(*w*), determined with a coverage factor of *k* = 2 are listed at each state point

*T*/K	*p*/MPa	*w*/m·s^−1^	100·*U*_r_ (*w*)
240.008	8.148	960.06	0.034
245.013	13.822	965.26	0.034
250.012	19.482	970.57	0.034
255.004	25.103	975.88	0.034
250.013	0.917	887.05	0.035
255.005	6.083	892.93	0.035
259.998	11.208	898.71	0.035
265.006	16.299	904.35	0.035
270.002	21.338	909.89	0.035
259.994	0.567	845.61	0.036
265.003	5.399	851.77	0.036
270.001	10.201	857.87	0.036
275.005	14.974	863.84	0.036
280.010	19.712	869.69	0.035
285.011	24.435	875.53	0.035
280.008	0.714	768.88	0.039
285.011	4.947	775.66	0.038
290.013	9.152	782.23	0.038
295.009	13.320	788.61	0.038
300.009	17.466	794.84	0.037
305.006	21.576	800.89	0.037
310.008	25.659	806.80	0.036
279.994	0.170	765.46	0.039
284.997	4.142	770.91	0.039
290.001	8.210	776.88	0.038
295.000	12.249	782.72	0.038
300.005	16.261	788.43	0.037
305.003	20.229	793.95	0.037
300.007	0.539	692.25	0.043
305.006	4.198	699.45	0.042
310.008	7.846	706.46	0.041
315.001	11.470	713.27	0.041
320.010	15.084	719.88	0.040
325.016	18.673	726.31	0.039
330.007	22.230	732.55	0.039
335.008	25.697	738.26	0.038
300.004	0.336	690.81	0.043
305.003	3.900	697.42	0.042
310.007	7.459	703.93	0.042
315.001	10.996	710.28	0.041
320.007	14.522	716.48	0.040
325.008	18.025	722.54	0.040
329.999	21.502	728.45	0.039
320.007	0.845	620.42	0.049
325.012	4.033	628.23	0.048
330.005	7.197	635.69	0.047
335.008	10.281	642.44	0.045
340.012	13.385	649.18	0.044
345.017	16.479	655.75	0.043
320.008	0.581	618.24	0.049
320.007	0.580	618.24	0.049
325.005	3.679	625.46	0.048
329.999	6.768	632.50	0.047
335.000	9.734	638.51	0.046
340.004	12.769	644.90	0.045
345.009	15.805	651.24	0.044
350.006	18.826	657.44	0.043
340.001	0.330	541.66	0.060
345.008	1.938	539.25	0.060
350.006	4.545	547.23	0.058
355.010	7.136	554.55	0.056
359.995	9.712	561.78	0.054
364.997	12.289	568.69	0.052
369.990	14.853	575.38	0.051
374.993	17.401	581.80	0.050
359.992	1.052	474.70	0.076
364.995	3.310	483.11	0.072
369.989	5.568	491.17	0.069
374.993	7.814	498.77	0.066
380.020	10.067	506.12	0.063
385.011	12.296	513.13	0.061
390.040	14.536	519.94	0.059
395.039	16.753	526.47	0.057
400.047	18.964	532.79	0.055
380.018	1.509	404.35	0.103
385.011	3.371	413.14	0.097
390.041	5.253	421.58	0.091
395.040	7.119	429.48	0.086
400.049	8.989	437.07	0.082
405.058	10.858	444.34	0.078
410.057	12.720	451.32	0.075
415.074	14.586	458.08	0.071
420.077	16.435	464.52	0.069
400.047	2.224	336.61	0.148
405.057	3.743	346.11	0.137
410.056	5.256	354.45	0.128
415.075	6.782	362.65	0.120
420.079	8.307	370.59	0.113

**Table 4:** Binary interaction parameters for the multifluid model estimated by REFPROP version 10.0 [9]

Parameter	Value
*β*_T_	1.00000
*β*V	1.00000
*γ*_T_	0.99072
*γ*_V_	1.01180

## Data Availability

The data reported in this study are deposited at nist.data.gov (DOI: https://doi.org/10.18434/mds2-3400).
